# Unique pathways downstream of TLR-4 and TLR-7 activation: sex-dependent behavioural, cytokine, and metabolic consequences

**DOI:** 10.3389/fncel.2024.1345441

**Published:** 2024-02-13

**Authors:** Isobel K. Dunstan, Ross McLeod, Daniel E. Radford-Smith, Wenzheng Xiong, Trinity Pate, Fay Probert, Daniel C. Anthony

**Affiliations:** ^1^Medical Sciences Division, Department of Pharmacology, University of Oxford, Oxford, United Kingdom; ^2^Department of Chemistry, Mathematical, Physical and Life Sciences Division, University of Oxford, Oxford, United Kingdom

**Keywords:** metabolomics, SARS-CoV-2, PAMP, Interleukin-1, N-acetyl aspartate

## Abstract

**Introduction:**

Post-infection syndromes are characterised by fatigue, muscle pain, anhedonia, and cognitive impairment; mechanistic studies exploring these syndromes have focussed on pathways downstream of Toll-like receptor (TLR) 4 activation. Here, we investigated the mechanistic interplay between behaviour, metabolism, and inflammation downstream of TLR-7 activation compared to TLR-4 activation in male and female CD1 mice.

**Methods:**

Animals received either a TLR-4 (LPS; 0.83 mg/kg) or TLR-7 (R848, 5 mg/kg) agonist, or saline, and behaviour was analysed in an Open Field (OF) at 24 h (*n* = 20/group). Plasma, liver, and prefrontal cortex (PFC) were collected for gene expression analysis at 24 h and 1H-NMR metabolomics.

**Results:**

TLR-4 and TLR-7 activation decreased distance travelled and rearing in the OF, but activation of each receptor induced distinct cytokine responses and metabolome profiles. LPS increased IL-1β expression and CXCL1 in the PFC, but TLR7 activation did not and strongly induced PFC CXCL10 expression. Thus, TLR7 induced sickness behaviour is independent of IL-1β expression. In both cases, the behavioural response to TLR activation was sexually dimorphic: females were more resilient. However, dissociation was observed between the resilient female mice behaviour and the levels of gene cytokine expression, which was, in general, higher in the female mice. However, the metabolic shifts induced by immune activation were better correlated with the sex-dependent behavioural dimorphisms; increased levels of antioxidant potential in the female brain are intrinsic male/female metabolome differences. A common feature of both TLR4 and TLR7 activation was an increase in N-acetyl aspartate (NAA) in the PFC, which is likely be an allostatic response to the challenges as sickness behaviour is inversely correlated with NAA levels.

**Discussion:**

The results highlight how the cytokine profile induced by one PAMP cannot be extrapolated to another, but they do reveal how the manipulation of the conserved metabolome response might afford a more generic approach to the treatment of post-infection syndromes.

## 1 Introduction

The risk of developing neuropsychiatric conditions, such as anxiety and mood disorders is increased following viral infection, and the recent COVID-19 pandemic has highlighted this phenomenon; one in eight COVID-19 patients received a neuropsychiatric diagnosis within 6 months of infection (Taquet et al., 2021). Many aspects of the reported post-viral syndromes are consistent with the adaptive sickness behaviours that are exhibited by both humans and rodents in the aftermath of a bacterial or viral infection, which are associated with an alteration in mood, anxiety, and cognition (Dantzer et al., 2008). They are typically transient and designed to assist in the recovery process and are clearly evident in individuals with SARS-CoV-2 infection (Davis et al., 2021). Once an infection has been successfully cleared, most individuals and animals gradually return to their normal behaviours and levels of activity. However, the specific duration and persistence of sickness behaviours can vary depending on the circumstances and the nature of the infection or illness and we, as have others, have shown that changes in behaviour in rodents persist beyond the acute phase in a manner that might be relevant to long COVID (Couch et al., 2015).

To date, the activation of Toll-Like Receptor (TLR)-4 by the endotoxin lipopolysaccharide (LPS) has been a prominent focus in the study of sickness behaviour in rodents owing to its central role in gram-negative bacterial recognition, but other TLRs also have the capacity to induce behavioural changes when activated (Cunningham et al., 2007; Yates et al., 2022). RNA viruses, such as SARS-CoV-2, are detected by TLR-7, a pattern recognition receptor (PRR) that recognises single-stranded RNA (ssRNA) (Lund et al., 2004; Salvi et al., 2021). The inflammatory response to TLR-7 stimulation induces a type-1 interferon response (Lund et al., 2004) and is initiated by the transcription factor IRF7. Activation of TLR-7 triggers a MyD88-dependent cascade, involving interleukin-1 receptor-associated kinase 1 (IRAK-1), IRAK-4, and TNF receptor-associated factor 6 (TRAF6), which leads to NFkB and IRF7 activation (Cao et al., 1996; Deng et al., 2000; Suzuki et al., 2002; Kawai et al., 2004), which, on the face of it, ought to generate a similar cytokine and, downstream, metabolic response to TLR-4 activation that also activates a MyD88-dependent pathway (Hemmi et al., 2002). However, the differences in the underlying mechanisms are large enough to justify exploration of the consequence of a TLR7-mediated event.

A further, often overlooked issue is that most of the LPS-based rodent studies are performed in male mice. However, sex is known to have a profound effect on immunological outcomes (Klein and Flanagan, 2016). It is widely reported that the females generally respond more strongly to an inflammatory challenge, leading to overall better vaccine efficacies and faster clearance of pathogens with less severe resulting disease, for example (Cook, 2008). Concordantly, 80% of autoimmune disorders occur in females (Klein and Flanagan, 2016). These differences are largely attributed to the actions of sex steroids, the receptors of which are found on most leuocytes (Kadel and Kovats, 2018). However, germline and other factors must contribute to these outcomes as differences are found throughout different stages of life and can be resilient to gonadectomy (Smith-Bouvier et al., 2008). Despite the striking influence of sex on immunity, many studies fail to take this factor into consideration (Klein and Flanagan, 2016). This issue is especially important in the current study, as TLR-7 is an X-linked gene as is IRAK, which is also pertinent to this study (Fish, 2008). Differences in expression and the downstream responses are known to arise owing to incomplete X chromosome inactivation in females in leuocyte populations especially (Souyris et al., 2018). In certain contexts, this has been reported to result in differences in susceptibility to infections, autoimmune diseases, or responses to immunomodulatory therapies that target TLR-7 (Shen et al., 2010; Jenks et al., 2018; Spiering and de Vries, 2021).

Immunity and metabolism are inextricably linked, and the energetic demands following immune activation are heightened as a result of cell differentiation, proliferation, and the execution of effector functions (Jung et al., 2019). Shifts in central metabolism following peripheral immune activation have been proposed to contribute to the behavioural effects of LPS challenge and can be detected using 1H NMR metabolomics (Chan et al., 2020). The metabolome is defined as the collection of small molecules (<1.5 kDa) in a biofluid such as plasma or urine, or extracted from a tissue, such as liver or brain. Influenced by internal (e.g., genetics) and external (e.g., lifestyle) factors, it is the closest of the “omics” platforms to the phenotype and is therefore, not only useful in determining pathological mechanisms, but can also be manipulated to resolve any differences (Patti et al., 2012). In the current study, it is used to explore the behavioural outcomes of immunological challenge.

Here, we sought to discover how TLR-7 activation results in sickness-associated behaviours in comparison to TLR-4 activation. We included males and females in the study design to investigate a sex-specific relationship between inflammation, metabolism, and behaviour.

## 2 Materials and methods

### 2.1 Animals and treatments

All procedures were carried out in accordance with the UK Animals (Scientific Procedures) Act 1986, and were approved by the Animal Welfare and Ethical Review Body of the University of Oxford. Animals were kept in standard laboratory housing conditions (12 h light/dark cycle, lights on at 7am; 21 ± 1°C; humidity 50 ± 5%) with access to both food and water *ad libitum*. Male and female 8-week-old CD1 mice (26-32 g; Charles River, UK) were administered i.p with R848 (5 mg/kg, ApexBio, Cat No. B1054), LPS (0.83 mg/kg, Sigma Aldrich) from Escherichia coli serotype O111:B4, or an equivalent volume of sterile saline.

### 2.2 Behaviour

The Open Field Test (OFT) was used to measure behaviour at 20 h post-injection, prior to tissue collection. The OFT apparatus consisted of four quadrants of equal dimensions (l × w × h; 50 cm × 50 cm × 50 cm) to enable four mice to be tested simultaneously. The open field was defined as 25% of the total area of each quadrant (l × w; 25 cm × 25 cm). Mice were placed in a randomised order in the top left corner of each quadrant and were video recorded for 5 min. 75% ethanol was used to wipe the floors and walls in between experiments to prevent the influence of odorants and coprophagy on mouse behaviour. ANY-Maze software (Stoelting Co., IL, USA) was used to measure all behavioural parameters except rearing, which was counted manually whilst blinded and was defined as any time a mouse was raised on its two hindlegs (supported by walls or free-standing).

### 2.3 Tissue collection

All animals were anaesthetised under isoflurane gas at 24 h post-injection. After checking for the absence of a pedal reflex, cardiac puncture was used to collect whole blood in EDTA (0.1 M, pH8)-coated tubes and centrifuged after 30 min at RT (10 min, 2000xg). Tissue was transcardially perfused with heparinised saline until the liver was clear of blood. Whole brains were extracted and snap-frozen in isopentane on dry ice and liver samples were collected and immediately snap-frozen on dry ice. All tissue and plasma was stored at −80°C for subsequent gene expression and metabolomics analysis.

### 2.4 RNA extraction and reverse transcription

RNA was isolated from 10 to 15 mg of the left prefrontal cortex (PFC) and liver samples according to manufacturer’s instructions [RNeasy^®^ Mini Kit (Cat No./ID: 74106)]. Liver and PFC RNA was eluted in nuclease-free water and 1000 ng was converted to cDNA with the Applied Biosystems High Capacity cDNA conversion kit, following manufacturer’s instructions. The reverse transcription step was carried out using the High-Capacity cDNA Reverse Transcription Kit (Thermofisher, cat. 4368813) using manufacturer’s instructions.

### 2.5 qPCR

Real-time qPCR was performed with samples in duplicate (25 ng/well) using the SsoAdvanced Universal SYBR Green Supermix (BioRad) with the Roche LightCycler 480. Transcripts of the acute phase response have previously been shown to be translated to protein (Campbell et al., 2003). Upon reaction completion, melt curve analysis was carried out to ensure primer specificity. The Ct values were normalised to the housekeeping gene, GAPDH, and were represented as the fold change relative to saline controls using the delta-delta CT (2^–Δ^ ^Δ^ ^CT^) method. Primer sequences can be found in Table 1.

**TABLE 1:** Primer sequences for qPCR.

Target gene	Primer sequences
BDNF	F: GGATATTGCGAAGGGTTATTAGATT
	R: GGAAGGTAATGTGTCTTGTTTGAA
CXCL-1	F: GCTGGGATTCACCTCAAGAAC
	R: TGTGGCTATGACTTCGGTTTG
CXCL-10	F: CATCCCGAGCCAACCTTCC
	R: CACTCAGACCCAGCAGGAT
GAPDH	F: AACGACCCCTTCATTGAC
	R: TCCACGACATACTCAGCAC
IL-1B	F: CAACCAACAAGTGATATTCTCCAT
	R: GGGTGTGCCGTCTTTCATTA
TLR4	F: CTGGCTAGGACTCTGATCATG
	R:GCATTGGTAGGTAATATTAGGAACTA
TLR7	F: GTACCAAGAGGCTGCAGATTAGAC
	R: AGCCTCAAGGCTCAGAAGATG
SAA1	F: CCATGCTCGGGGGAACTATG
	R: GCCCCCAGCACAACCTACT
SAA-2	F: TGGCTGGAAAGATGGAGACAA
	R: AAAGCTCTCTCTTGCATCACTG
SAA3	F: CTGTTCAGAAGTTCACGGGAC
	R: AGCAGGTCGGAAGTGGTT

### 2.6 1H NMR metabolomics

#### 2.6.1 Sample processing

Metabolites were extracted from brain and liver samples as previously described (Radford-Smith et al., 2022). Briefly, a pestle and mortar was used to homogenise 100 mg (± 10%) fresh tissue on dry ice before adding 8 uL/mg sample of ice-cold acetonitrile (50%). Samples were then vortexed for mixing and centrifuged at 5060 × *g* for 5 min at 4°C. Supernatants were snap frozen on dry ice, lyophilised overnight and stored at −80°C until the day of NMR. When ready for analysis, lyophilised samples were placed at room temperature and resuspended in 550 uL of NMR buffer (75 mM sodium phosphate buffer prepared in D2O, pH 7.4). The plasma samples were also allowed to thaw at RT and 150 uL was added to 400 uL NMR buffer.

#### 2.6.2 NMR experiments

All prepared samples were then placed in a 5 mm NMR tube and measured using a 700-MHz Bruker AVII spectrometer operating at 16.4 T, equipped with a 1H (13C/15N) TCI cryoprobe, as described previously (Chan et al., 2020). Plasma and liver spectra were acquired using a spin-echo sequence (Carr-Purcell-Meiboom-Gill [CPMG]), 32 data collections, an acquisition time of 1.5 s, a relaxation delay of 2 s, and a fixed receiver gain. Brain spectra were acquired with a Nuclear Overhauser Effect Spectroscopy (NOSEY) sequence as previously described (Chan et al., 2020). All samples were run within 9-h of being thawed.

#### 2.6.3 NMR data processing

Spectra were phased, baseline-corrected and referenced to the lactate doublet at δ = 1.33 ppm in Topspin 4.1.4 (Bruker). ACD/Labs Spectrus Processor Academic Edition 12.01 (Advanced Chemistry Development, Inc.) was used to manually bin each resonance signal, excluding all noise from analysis. The integrals of these bins were sum normalised and exported to R. Metabolites assignment was conducted through a mix of in-house databases, literature reviews, and 2D total correlation spectroscopy (TOCSY) experiments.

### 2.7 Statistical analysis

#### 2.7.1 Univariate

All statistical analyses were carried out using the GraphPad Prism 9 software. Any outliers were identified using the GraphPad Outlier Calculator and were removed accordingly. The Shapiro-Wilk normality test was used to test for normality and log transformation was applied for the statistical analysis in instances that the test was not passed. Two-way analysis of variance (ANOVA) was used to assess a main effect of treatment (TLR agonist administration) on behaviour, relative mRNA expression, and spectral intensity. *Post-hoc* Tukey’s tests were then used for multiple comparisons if there was a significant interaction between treatment and sex. The significance threshold was set to *p < 0.05, **p < 0.01, ***p < 0.001, ****p < 0.0001 and all results were presented as mean ± standard error of the mean (SEM). All correlations were performed via Pearson correlational analyses and were corrected for false discovery rate (FDR).

#### 2.7.2 Multivariate

The ROPLS package in R was used for orthogonal partial least squares discriminant analysis (OPLS-DA) of the metabolomes to determine whether there were any between group differences. Variable importance in projection (VIP) scores were used to detect which metabolites were most important for driving separation between groups. Bins with highest VIP scores (before a point of inflexion when comparing all VIP scores) then underwent univariate analyses to understand the direction of change of metabolites, as described above.

## 3 Results

### 3.1 Female mice are resistant to toll-like receptor (TLR)-induced sickness behaviour

**FIGURE 1:**
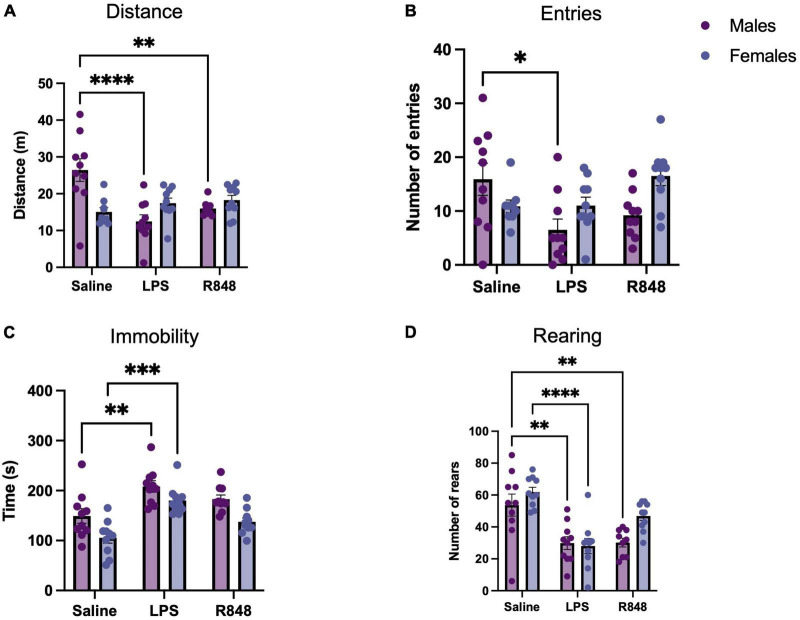
Toll-like receptor (TLR) activation caused sickness behaviour in mice 24 h post-injection. **(A)** Distance travelled during a 5-min open field test (OFT), **(B)** Number of entries to the centre zone, **(C)** Total time spent immobile, **(D)** Number of rears. Data represented as mean ± SEM, *p < 0.05, **p < 0.01, ***p < 0.001, ****p < 0.0001 indicating significant *post hoc* Tukey’s comparisons following a significant main effect of TLR activation as determined by two-way ANOVA, *n* = 9-10/group.

At 24 h, following the administration of the TLR-7 agonist R848 or the TLR-4 agonist LPS, two-way ANOVA confirmed that there was a main effect of treatment on the distance travelled in the OFT [Figure 1A, *F*_(2, 52)_ = 5.367, *p* = 0.0076] and that there was a significant interaction between treatment and sex [*F*_(2, 52)_ = 12.02, *p* < 0.0001]. However, *post-hoc* Tukey’s testing revealed that while both LPS and R848 treatment decreased the distance travelled in the OFT in male mice (LPS *p* < 0.001; R848 *p* < 0.01), distance travelled was not reduced in female mice (LPS *p* = 0.93; R848 *p* = 0.80). Further analysis of the OFT data revealed that there was also a main effect of treatment on the number of entries into the centre zone of the open field [Figure 1B, *F*_(2, 51)_ = 3.5187, *p* = 0.0368] and, again, a significant interaction between treatment and sex [*F*_(2, 53)_ = 5.5, *p* = 0.0064]. Tukey’s *post hoc* comparisons revealed that the number of entries into the centre zone was decreased by LPS only in males (*p* < 0.001). Two-way ANOVA also revealed a significant effect of treatment on total immobility time and the total number of exploratory rears during the OFT [immobility *F*_(2, 54)_ = 20, *p* < 0.0001; rearing *F*_(2, 53)_ = 9.672, *p* < 0.001]. For these parameters there was no significant interaction between treatment and sex, but this result reveals that the female mice also exhibit sickness behaviours in response to the challenges (Figures 1C, D).

### 3.2 The classical TLR-4 induced cytokine response is absent following TLR-7 activation despite the induction of sickness behaviours

**FIGURE 2:**
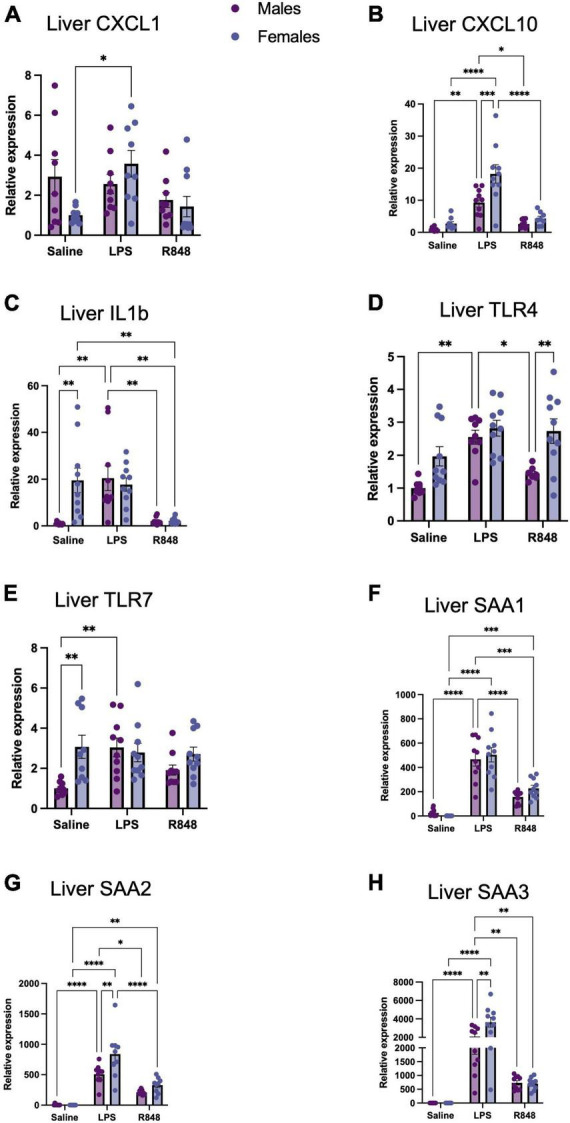
TLR-4 and TLR-7 activation by LPS and R848, respectively, increased expression of inflammatory genes in the liver 24 h post-injection. Relative expression of hepatic **(A)** CXCL-1, **(B)** CXCL-10, **(C)** IL-1b, **(D)** TLR-4, **(E)** TLR7, **(F)** SAA1, **(G)** SAA2, and **(H)** SAA3 mRNA. Results are presented as mean ± SEM, *p < 0.05, **p < 0.01, ***p < 0.001, ****p < 0.0001 indicating significant *post hoc* Tukey’s comparisons following a significant main effect of TLR activation as determined by two-way ANOVA, *n* = 9-10/group.

**FIGURE 3:**
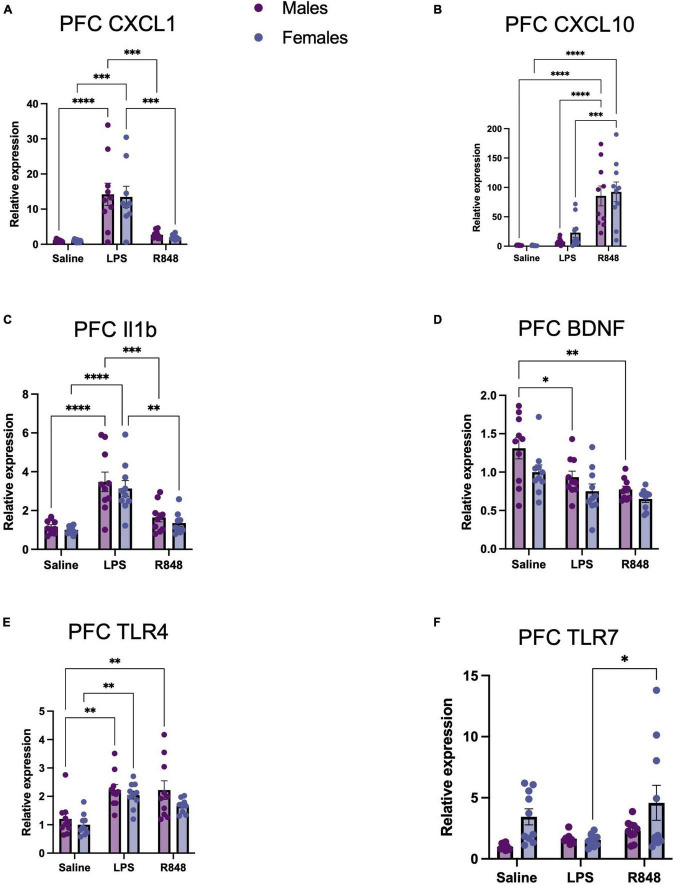
TLR-4 and TLR-7 activation by LPS and R848, respectively, increased expression of inflammatory genes in the prefrontal cortex (PFC) 24 h post-injection. Relative expression of **(A)** CXCL-1, **(B)** CXCL-10, **(C)** IL-1b, and **(D)** BDNF, **(E)** TLR4, and **(F)** TLR7 mRNA. Results are presented as mean ± SEM, *p < 0.05, **p < 0.01, ***p < 0.001, ****p < 0.0001 indicating significant *post hoc* Tukey’s comparisons following a significant main effect of TLR activation as determined by two-way ANOVA, *n* = 9–10/group.

TLR-4 and TLR-7 activation induced the mRNA expression of proinflammatory genes both in the liver, as part of the acute phase response (Figure 2), and in the prefrontal cortex (Figure 3) in male and female mice. Two-way ANOVA revealed a main effect of treatment on hepatic CXCL-1, CXCL-10, and IL-1β expression [*F*_(2, 50)_ = 3.964, *p* = 0.0252, *F*_(2, 51)_ = 41.71, *p* < 0.0001, and *F*_(2, 54)_ = 13.57, *p* < 0.0001, respectively] and, for these transcripts, there was a significant interaction between treatment and sex [CXCL1 *F*_(2, 50)_ = 3.682, *p* = 0.0322; CXCL10 *F*_(2, 51)_ = 4.313, *p* = 0.0186; IL1b *F*_(2, 54)_ = 6.248, *p* = 0.0036]. Somewhat counterintuitively, Tukey’s *post hoc* comparisons revealed that hepatic CXCL-1 expression was elevated relative to controls only in females 24 h post-LPS injection (*p* < 0.001). CXCL-10 expression was raised at 24 h following LPS in both males (*p* < 0.01) and females (*p* < 0.0001), although significantly higher in females (*p* < 0.001), whereas liver IL-1β was higher in females at baseline (*p* < 0.01) and thus, only induced in males by LPS (*p* < 0.01). Interestingly, there was no significant effect of R848 on the expression of CXCL1 or CXCL10, in males or in females and IL-1β was lower in female R848 mice alone relative to controls. Two-way ANOVA revealed a main effect of treatment on receptor expression in the liver [TLR-4 *F*_(2, 50)_ = 11.69, *p* < 0.0001; TLR-7 *F*_(2, 53)_ = 2.661, *p* = 0.0792] and an interaction between treatment and sex for TLR-7 alone [*F*_(2, 53)_ = 4.384, *p* = 0.0173]. *Post hoc* analysis indicated that females have a higher baseline expression of TLR-7 in the liver compared to males (*p* < 0.01) yet no increase in expression following either challenge in females. Oddly, LPS increased TLR-7 expression in males (*p* < 0.01), in a similar manner to the expression of IL-1β. Both challenges induced three isoforms of the classical acute phase protein, Serum Amyloid A (SAA) [N.B. SAA is an apolipoprotein that is known to bind LPS (Cheng et al., 2018)] [SAA1 *F*_(2, 54)_ = 90.16 *p* < 0.0001; SAA2 *F*_(2, 48)_ = 61.69 *p* < 0.0001; SAA3 *F*_(2, 54)_ = 65.47 *p* < 0.0001]. Furthermore, there was a significant interaction between treatment and sex for SAA2 and SAA3 [*F*_(2, 48)_ = 3.94, *p* = 0.026 and *F*_(2, 54)_ = 6.101, *p* = 0.0041, respectively], with females inducing a greater acute phase response (APR) following LPS in both cases (*p* < 0.01).

In the brain, two-way ANOVA revealed a main effect of treatment on the mRNA expression of proinflammatory genes in the prefrontal cortex 24 h post-challenge [Figure 3, CXCL1 *F*_(2, 52)_ = 31.31, *p* < 0.0001; CXCL10 *F*_(2, 54)_ = 42.04, *p* < 0.0001; IL-1β *F*_(2, 53)_ = 31.85, *p* < 0.0001; BDNF *F*_(2, 53)_ = 12.66, *p* < 0.0001], but there were no significant interactions. However, both challenges provoked a different pattern of cytokine/chemokine expression. As confirmed by *post hoc* Tukey’s analysis, TLR-4 activation by LPS increased prefrontal cortex CXCL-1 (males *p* < 0.001; females *p* < 0.0001) and IL-1β expression (males *p* < 0.0001, females *p* < 0.0001) at 24 h, but did not alter CXCL10 expression. Conversely, TLR-7 activation by R848 increased prefrontal cortex CXCL-10 expression (male *p* < 0.0001; female *p* < 0.0001) and did not significantly affect the expression of CXCL1 or, intriguingly, IL-1β. Checking receptor expression in this brain region revealed a main effect of treatment on TLR-4 [*F*_(2, 54)_ = 16.36, *p* < 0.0001] and TLR-7 [*F*_(2, 53)_ = 3.907, *p* < 0.05] and no interactions between LPS and sex. It was of interest to note, that no male/female differences were detectable for these transcripts. As a consequence, we sought to discover whether metabolic changes in the blood, liver and brain might underpin the observed differences in behaviour.

### 3.3 TLR-4 and TLR-7 activation give rise to distinct metabolic signatures in the brain, plasma, and liver

**FIGURE 4:**
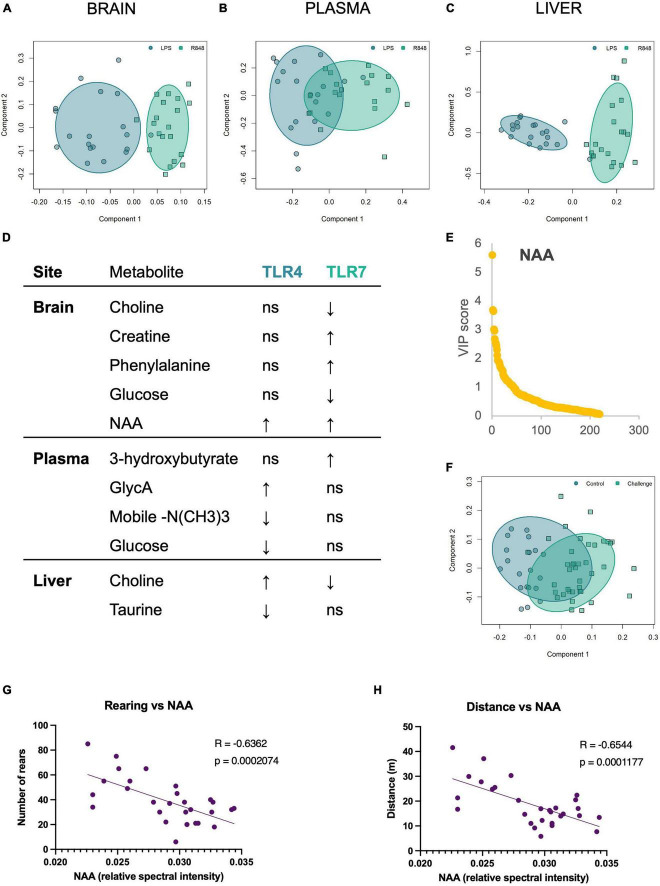
TLR-4 and TLR-7 activation gave rise to distinct metabolome profiles, yet both increased brain NAA, which correlated with the sickness behaviour displayed by males. OPLS-DA scores plot of **(A)** brain, **(B)** plasma, and **(C)** liver metabolites demonstrate that the metabolic shift following immune activation depends on the TLR agonist. **(D)** The direction of change of the discriminatory metabolites for each tissue site, relative to saline controls. Metabolites were selected based on Variable Importance in Projection (VIP) scores. **(E)** VIP plot and **(F)** associated OPLSDA scores plot of the combined TLR groups (“challenge”) vs. saline control brain metabolites, confirming that brain NAA consistently increases following TLR activation, and scores as the most discriminatory metabolite between inflammatory challenge and control. **(G)** Pearson correlation of rearing behaviour and **(H)** distance travelled in the OFT by males against brain NAA levels. All groups were composed of 50/50 male/female CD1 mice. Individual box plots for these key metabolites are all shown in Supplementary Figure 1 (brain), Supplementary Figure 2 (plasma), and Supplementary Figure 3 (liver).

Analysis of the metabolomic data from aqueous NMR spectra using supervised orthogonal partial least squares discriminant analysis (OPLS-DA) revealed the presence of distinct metabolic profiles for the groups treated with R848 and LPS for all tissue types (Figure 4). A range of metabolites differed, reflecting the impact of the precise nature of inflammation on host metabolism. Figure 4D summarises the direction of change of the most discriminatory metabolites relative to the saline controls and the box plots for metabolites are presented in Supplementary Figures 1–3. It was of interest to note that brain N-acetyl aspartate (NAA) was a conserved marker of TLR challenge, increasing in the LPS and R848 groups relative to controls. To determine whether this change could account for the shared behavioural response in males, we performed a Pearson correlational analysis (Figures 4G, H). Indeed, brain NAA was found to correlate highly with distance (*r* = −0.63, *p* = 0.0003) and rearing (*r* = −0.59, *p* = 0.0007), indicating that this conserved central marker of a peripheral TLR inflammatory challenge is associated with stereotypical sickness behaviours.

### 3.4 Males and females have a distinct metabolic response to TLR challenge that is driven by changes in the brain antioxidant system and is associated with behaviour

**FIGURE 5:**
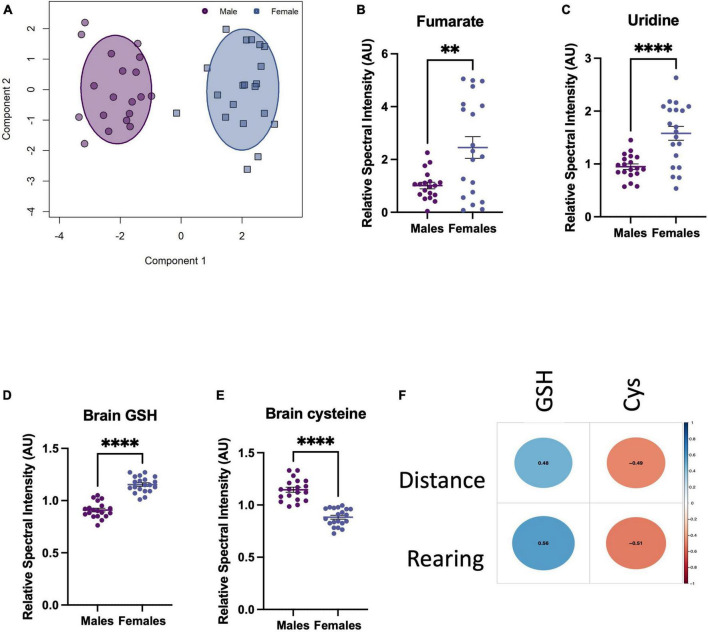
Toll-like receptor (TLR) challenge induces a sex-dependent metabolic response in the brain 24 h after i.p. TLR agonist administration. **(A)** OPLS-DA scores plot of male vs. female brain metabolites after a TLR challenge, normalised to their respective male/female saline control metabolites. Normalisation avoided the influence of baseline sex differences in brain metabolomes, as we were interested in how males and females respond differently to the TLR challenge. **(B–E)** Top VIP metabolites, selected before a point of inflexion when comparing all VIP scores. These metabolites are the most discriminatory between the male vs. female inflammatory metabolomes and are presented normalised to baseline, i.e., male challenge vs. female challenge (TLR4 and TLR7 groups combined)/saline bins. **(F)** Pearson correlational analysis between sickness behaviours displayed by males and the corresponding GSH an Cys levels in the brain, *R*-values shown in the centre of each circle. Data presented as mean ± SEM, *p < 0.05, **p < 0.01, ***p < 0.001, ****p < 0.0001.

Given that females displayed more resistant behaviour to the TLR challenges, we sought to explore whether the brain metabolic response to challenge depended on sex. First, brain metabolite bins of TLR-challenged males and females were normalised to their corresponding saline control groups to avoid identifying baseline sex differences in the metabolome. OPLS-DA of these normalised groups revealed that the metabolic changes resulting from TLR challenge were distinct for males and females (Figure 5A). Variable importance in projection (VIP) scores revealed the most discriminatory metabolites were brain fumarate, uridine, glutathione (GSH) and cysteine (Cys) (Figures 5B–E). Of these discriminatory metabolites, the distribution of GSH and its precursor, Cys, were particularly distinct and non-overlapping between sexes and were found to strongly correlate with behaviour (Figure 5F). The increase in female antioxidant response in the brain could therefore, account for the resilient behavioural phenotype.

## 4 Discussion

Here we sought to compare the acute phase response and changes to the metabolome downstream of systemic TLR-4 and TLR-7 activation in male and female mice. Analysis of the sickness behaviours induced by TLR challenges revealed that the female mice, compared to their male counterparts, were more resilient but cytokine production in the brain and liver in the females was either the same or elevated compared to the males. Given that the central proinflammatory cytokine response is viewed as a dose-dependent regulator of sickness behaviour this was an unexpected result. However, perhaps more striking, were the distinct metabolomic and cytokine responses to each challenge. TLR7-induced sickness behaviour was independent of increased central IL-1β expression. The metabolic shifts induced by immune activation were better correlated with the sex-dependent behavioural dimorphisms; increased levels of antioxidant potential in the female brain are likely to govern the intrinsic male/female metabolome differences. Among the distinct challenge-specific changes observed, a notable common feature of both TLR4 and TLR7 activation were marked changes in NAA in the PFC that were inversely correlated with the sickness behaviours. These observations will be discussed in turn below.

Female mice have been shown in a few cases to be more resilient, in terms of behaviour, to an LPS challenge (Millett et al., 2019). Previous studies of LPS challenge in CD1 mice have similarly found females to demonstrate more resilient behaviours in terms of recovery time (Meneses et al., 2018). These studies also identified more profound cytokine responses in females, further demonstrating an inconsistency in the classical paradigm of inflammation-induced negative affect (Cai et al., 2016; Murray et al., 2019). In the current study, we found that female resilience was accompanied by a more marked hepatic acute phase response (SAA2/3 and CXCL10) following the administration of LPS especially. The increase in basal TLR7 and IL-1β expression in the females is likely to be a consequence of the location of TLR-7 on the X-chromosome. Biallelic B lymphocytes from women display greater TLR7 transcriptional expression than the monoallelic cells, and this was correlated with higher TLR7 protein expression in female than in male leuocyte populations (Souyris et al., 2018). Thus, TLR7 escape from X inactivation, which endows the B cell compartment with added responsiveness to TLR7 ligands, might have been expected to generate greater responses in the female vs. the male mice. While this was true to some extent, it should be noted that hepatic IL-lβ expression was suppressed in the male and female mice in response to the TLR7 agonist, and, in the prefrontal cortex, despite the resilient behaviour exhibited by the female mice in response to TLR4 and TLR7 activation, the induction of each cytokine was comparable between sexes.

In addition to the observed sex-specific cytokine response, the type of TLR ligand gave rise to distinct cytokine profiles. Despite both TLR-4 and TLR-7 activation inducing sickness behaviour, the expression of prefrontal cortex (PFC) CXCL-1 and IL-1β was only elevated by the TLR-4 ligand, LPS. Conversely, PFC CXCL-10 was only altered by TLR-7 activation. Central IL-1β has long been recognised as a mediator of inflammation-associated behaviours, as direct injection of IL-1β into the brain generates sickness behaviour (reduced activity and exploratory behaviour, fever, and reduced food intake), while administration of the IL-1β receptor antagonist (IL- 1RA) reverses these behaviours (Kent et al., 1992). Thus, the induction of CNS IL-1β by LPS was not unexpected; we and many others have used a peripheral injection of LPS to induce central IL-1β expression over many years (Van Dam et al., 1992; Layd et al., 1994; Radford-Smith et al., 2023). Other studies have found a correlation between elevated levels of IL-1β in the blood or cerebrospinal fluid and the severity of sickness behaviour in animals (Dantzer, 2001). This further supports the idea that IL-1β is a key mediator of these behaviours. In the PFC mRNA encoding IL-1R1 and IL-1AcP has been reported to be present in pyramidal cells and interneurons and IL-1β was shown to induce excitatory/inhibitory imbalances at the network level (Mittli et al., 2023). In human studies, elevated levels of IL-1β have been observed in individuals with major depressive disorder and other mood disorders. These findings suggest a link between IL-1β and symptoms of depression, which are often part of sickness behaviour. Thus, it was a surprise that that TLR-7 activation, similar to TLR-4, induces stereotypical sickness behaviours but without the induction of PFC IL-1β. Together, these sex- and TLR-specific effects on behaviour suggest that the relationship between inflammation and behaviour is more complex and that other factors contribute to the behavioural effects of TLR challenge. Here CXCL10, rather than IL-1β, was strongly induced by R848, which we previously reported 6 h after an R848 challenge in male mice (Yates et al., 2022). Others have shown that at 24 h post-LPS injection, using a comparable dose of LPS, CCL2 and CXCL10 were elevated in the brain and CXCL10 expression in the brain was most positively and significantly correlated with sickness behaviour (Davis et al., 2017). More relevant to TLR7 activation, in mice challenged with vesicular stomatitis virus (VSV)-M2, CXCL10 from brain endothelia was shown to disrupt synaptic plasticity and generate virus-induced sickness behaviour (Blank et al., 2016). In their compelling paper, mice lacking CXCR3 or CXCL10 and subjected to IFN-β treatment or infection with the virus were protected from depressive-like behaviour. Thus, brain CXCL10, as for IL-1β, is clearly able to initiate sickness behaviours, but it remains unclear whether the action is directly on neurons or via an intermediate cell population such as microglia or astrocytes (Van Weering et al., 2011).

To explore potential unknown mediators of sickness behaviour, we turned to the metabolome as it is influenced by both intrinsic and extrinsic factors and is the closest of the omics to the phenotype. Surprisingly, TLR-4 and TLR-7 activation gave rise to distinct metabolic profiles in the liver, plasma, and brain at 24 h. Most notably, the decrease in plasma glucose following LPS administration was not observed following R848. Hypoglycaemia resulting from endotoxaemia is often recognised for its sickness-inducing properties (Chan et al., 2020), yet this shows this is not the cause of all post-infection related fatigue and mood alterations. Pertinently, following a more viral-like challenge by R848 compared to LPS, glucose remained unchanged and reveals the metabolic response is sensitive to the nature of the inflammatory challenge. Despite the accuracy of the OPLSDA models, indicating a clear separation between TLR-4 and TLR-7 groups, brain NAA increased consistently across all groups at 24 h post-injection and was the most discriminatory metabolite when comparing the TLR challenge (LPS and R848 groups combined) to controls. The role of NAA in the brain remains elusive; however, it is the precursor of the most abundant neuropeptide, NAAG, and may be involved in myelin synthesis and neuronal metabolism. It has been demonstrated to reach high concentrations in the brain following acute inflammatory insult and has been associated with white matter destruction in such cases (Moffett et al., 2008). Here, we show NAA is a non-specific marker of peripheral PRR activation, which associates with the sickness behaviour displayed by male mice.

Next, we sought to determine whether the brain metabolic response to TLR challenge depended on sex. After accounting for baseline sex differences, OPLS-DA analysis revealed the male “inflammatory” metabolome was distinct from that of the females. This shows that the males and females presented a divergent metabolic response to the challenge. While sex differences in immune responses have clearly been established in both rodents and humans, reports on sexual dimorphisms in immunometabolism are comparatively scarce. When testing the difference between the male and female brain metabolites after TLR challenge, we found comparatively lower brain glutathione (GSH) and respective raised cysteine (Cys) in the males. Depleted brain GSH has been used to model neuronal loss and cognitive decline in rodents (González-Fraguela et al., 2018), is associated with cognitive performance in humans and has been demonstrated, via magnetic resonance spectroscopy, to be higher in female than male brains (Mandal et al., 2012). The association between brain GSH and its precursor, Cys, with rearing and distance travelled in the OF could underly the sex-specific behavioural phenotypes in the current study. In humans, depression, anxiety, and cognitive decline have been associated with oxidative stress (Correia et al., 2023). Moreover, lower antioxidant potential in the brain following COVID-19 has been identified in those convalescing, illustrating that this may also be a phenomenon of post-viral mood disorders, and may explain the more resilient behaviours of the female mice in the current study (Al-Hakeim et al., 2023).

## 5 Conclusion

Males and females respond differently to an immune challenge. The current study demonstrates that the relationship between cytokines and behaviour is not dose-dependent but is sex-specific. This was demonstrated by the higher levels of cytokines induced by TLR-4 and TLR-7 activation in females relative to males, despite the more resilient behaviour demonstrated by females. Additionally, the cytokine and corresponding metabolic response is sensitive to the nature of the inflammatory challenge. Specifically, the LPS-induced IL-1β and glucose responses may not be extrapolated to other TLR challenges. The metabolic shift following TLR challenge was also dependent on the agonist, as well as sex. A striking commonality between the TLR challenges was the response of brain NAA, which was increased in both groups relative to saline controls and correlated strongly with the common sickness behaviours in the TLR male mice. Finally, most disparate changes in the brain metabolomes of male and female mice included a higher antioxidant response in females, which may underly behavioural resilience.

## Data availability statement

The original contributions presented in the study are included in the article/Supplementary material, further inquiries can be directed to the corresponding author.

## Ethics statement

The animal study was approved by the LERP and ACER, University of Oxford. The study was conducted in accordance with the local legislation and institutional requirements.

## Author contributions

ID: Data curation, Formal Analysis, Investigation, Methodology, Project administration, Writing – original draft. RM: Data curation, Project administration, Writing – review and editing. DR-S: Supervision, Writing – review and editing. WX: Data curation, Writing – review and editing. TP: Data curation, Writing – review and editing. FP: Supervision, Writing – review and editing. DA: Conceptualization, Supervision, Writing – review and editing.

## Supplementary material

Supplementary Figure 1 Brain metabolites that are most discriminatory between TLR4 and TLR7 groups.

Supplementary Figure 2 Liver metabolites that are most discriminatory between TLR4 and TLR7 responses.

Supplementary Figure 3 Plasma metabolites that are most discriminatory between TLR4 and TLR7 responses.

Supplementary Table 1 Primer sequences for qPCR.
