# Supplementary figures and images for: Unique pathways downstream of TLR-4 and TLR-7 activation: sex-dependent behavioural, cytokine, and metabolic consequences
Source: Front Cell Neurosci. 2024 Feb 13;18:1345441. doi: 10.3389/fncel.2024.1345441 (PMC10896997; doi:10.3389/fncel.2024.1345441)

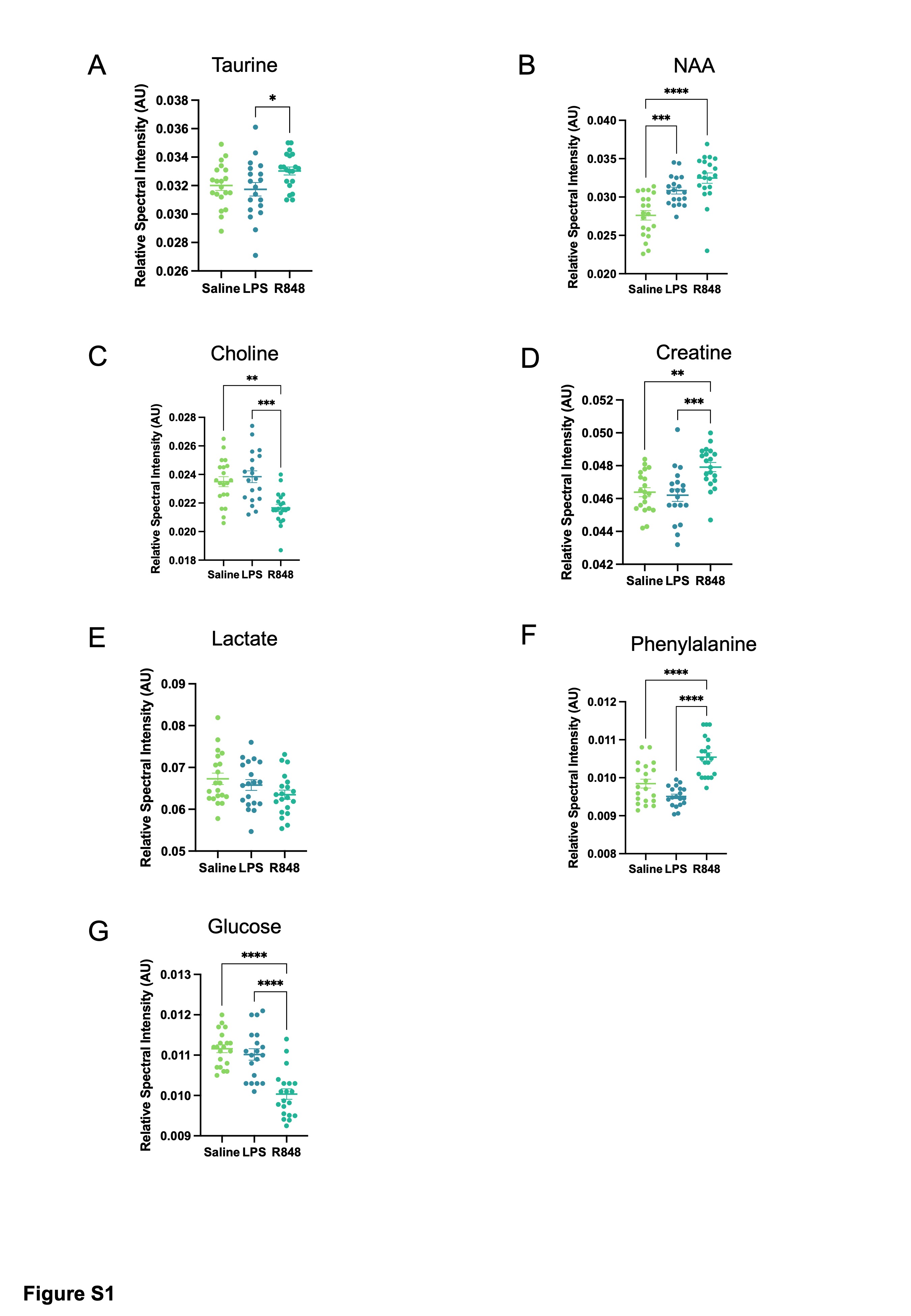

Supplement: Supplementary Figure 1 — Brain metabolites that are most discriminatory between TLR4 and TLR7 groups. [file Image_1.JPEG]

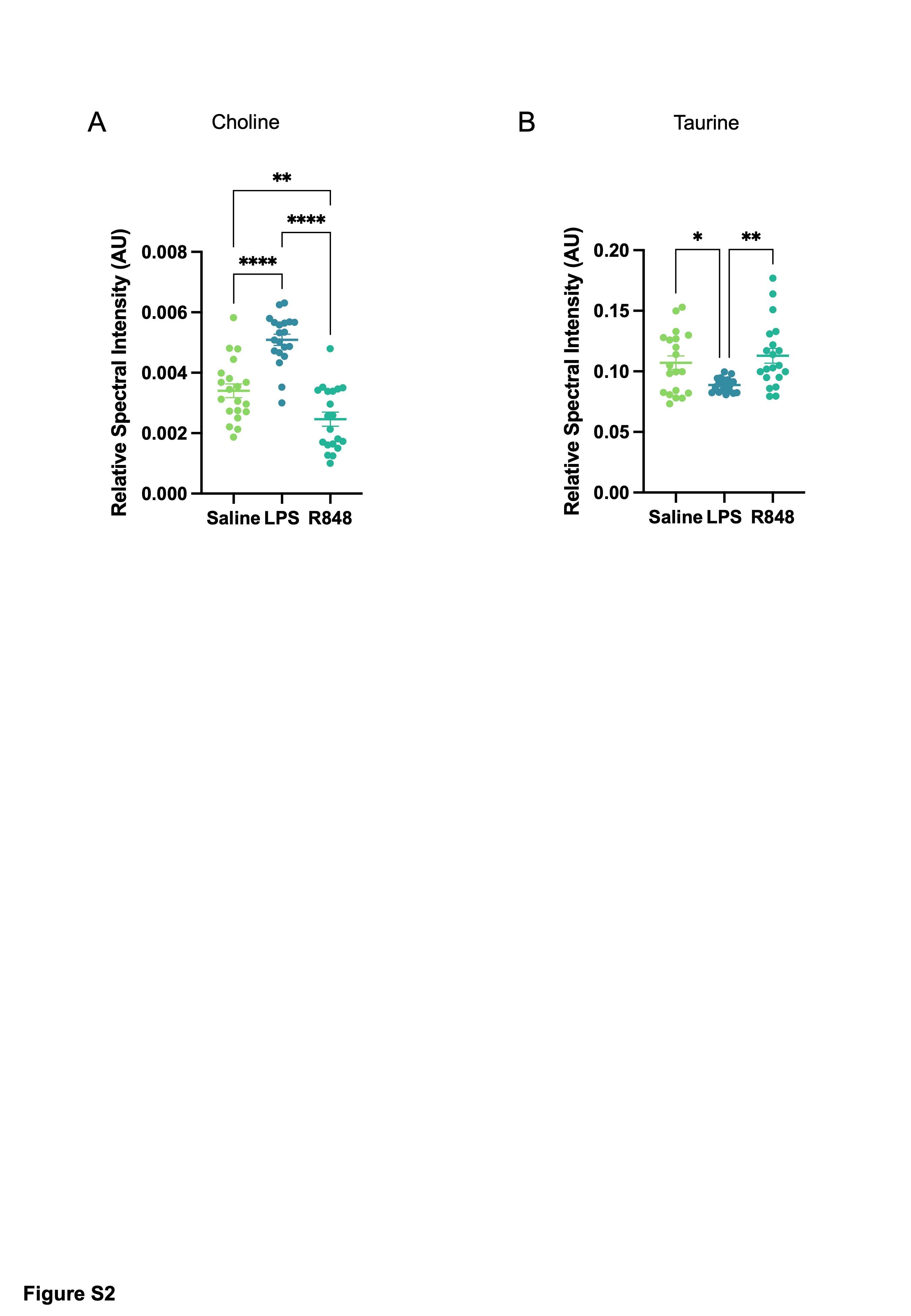

Supplement: Supplementary Figure 2 — Liver metabolites that are most discriminatory between TLR4 and TLR7 responses. [file Image_2.JPEG]

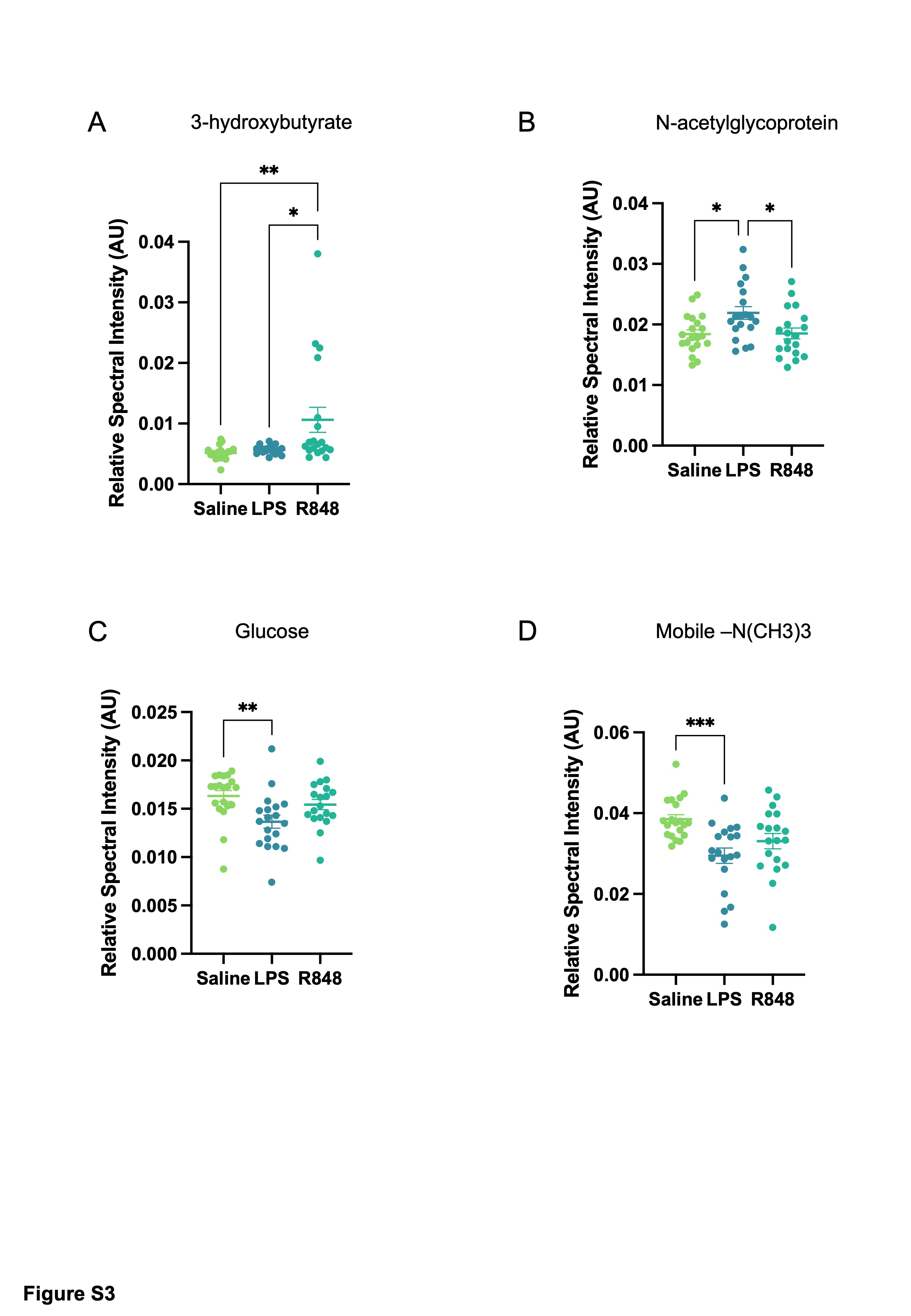

Supplement: Supplementary Figure 3 — Plasma metabolites that are most discriminatory between TLR4 and TLR7 responses. [file Image_3.JPEG]
