# Supplementary material for: Unique pathways downstream of TLR-4 and TLR-7 activation: sex-dependent behavioural, cytokine, and metabolic consequences
Source: Front Cell Neurosci. 2024 Feb 13;18:1345441. doi: 10.3389/fncel.2024.1345441 (PMC10896997; doi:10.3389/fncel.2024.1345441)
Supplement: Supplementary Table 1 — Primer sequences for qPCR. [file Table_1.DOCX]

|  | Males | | | Females | | |
| --- | --- | --- | --- | --- | --- | --- |
| **Distance** |  |  |  |  |  |  |
|  | Saline | LPS | R848 | Saline | LPS | R848 |
| Minimum | 5.82 | 1.241 | 13.98 | 11.9 | 7.789 | 11.95 |
| Maximum | 41.56 | 22.38 | 20.53 | 22.53 | 22.39 | 22.9 |
| Range | 35.74 | 21.14 | 6.554 | 10.63 | 14.6 | 10.96 |
| Mean | 26.44 | 12.49 | 15.98 | 15.05 | 17.44 | 18.28 |
| Std. Deviation | 9.796 | 5.661 | 2.03 | 3.503 | 4.312 | 4.068 |
| Std. Error of Mean | 3.098 | 1.79 | 0.6768 | 1.168 | 1.364 | 1.286 |
|  |  |  |  |  |  |  |
| **Entries** |  |  |  |  |  |  |
| Minimum | 0 | 0 | 3 | 6 | 1 | 7 |
| Maximum | 31 | 20 | 17 | 19 | 18 | 27 |
| Range | 31 | 20 | 14 | 13 | 17 | 20 |
| Mean | 15.9 | 6.5 | 9.2 | 10.89 | 11 | 16.5 |
| Std. Deviation | 9.386 | 6.346 | 4.185 | 3.516 | 4.989 | 5.603 |
| Std. Error of Mean | 2.968 | 2.007 | 1.323 | 1.172 | 1.578 | 1.772 |
|  |  |  |  |  |  |  |
| **Immobility** |  |  |  |  |  |  |
| Minimum | 87.6 | 162.9 | 147.9 | 51.3 | 152.7 | 99.6 |
| Maximum | 252.5 | 286.9 | 237 | 165 | 251 | 185.5 |
| Range | 164.9 | 124 | 89.1 | 113.7 | 98.3 | 85.9 |
| Mean | 149.2 | 208.3 | 183 | 105.6 | 180.1 | 137.4 |
| Std. Deviation | 46.71 | 36.09 | 25.91 | 34.49 | 28.66 | 24.61 |
| Std. Error of Mean | 14.77 | 11.41 | 8.195 | 10.91 | 9.064 | 7.782 |
|  |  |  |  |  |  |  |
| **Rearing** |  |  |  |  |  |  |
| Minimum | 6 | 9 | 18 | 49 | 2 | 30 |
| Maximum | 85 | 51 | 40 | 76 | 60 | 56 |
| Range | 79 | 42 | 22 | 27 | 58 | 26 |
| Mean | 53.7 | 29.9 | 30.22 | 61.9 | 28 | 46.9 |
| Std. Deviation | 21.93 | 12.62 | 8.318 | 9.315 | 15.07 | 8.875 |
| Std. Error of Mean | 6.936 | 3.99 | 2.773 | 2.946 | 4.766 | 2.807 |
|  |  |  |  |  |  |  |
| **Liver CXCL1** |  |  |  |  |  |  |
| Minimum | 0.4114 | 1.089 | 0.5298 | 0.5838 | 0.5798 | 0.3759 |
| Maximum | 7.483 | 5.384 | 4.18 | 1.663 | 6.447 | 4.785 |
| Range | 7.071 | 4.294 | 3.65 | 1.079 | 5.867 | 4.409 |
| Mean | 2.928 | 2.565 | 1.761 | 1 | 3.579 | 1.435 |
| Std. Deviation | 2.573 | 1.444 | 1.116 | 0.3606 | 2.001 | 1.613 |
| Std. Error of Mean | 0.8576 | 0.4812 | 0.3721 | 0.114 | 0.6672 | 0.5101 |
|  |  |  |  |  |  |  |
| **Liver CXCL10** |  |  |  |  |  |  |
| Minimum | 0.3265 | 1.039 | 1.214 | 1.227 | 2.028 | 1.821 |
| Maximum | 2.114 | 14.57 | 4.302 | 6.704 | 36.38 | 7.675 |
| Range | 1.788 | 13.53 | 3.088 | 5.477 | 34.35 | 5.853 |
| Mean | 1 | 9.287 | 2.606 | 2.747 | 18.21 | 4.304 |
| Std. Deviation | 0.5379 | 4.445 | 1.232 | 1.753 | 9.082 | 2.061 |
| Std. Error of Mean | 0.1701 | 1.406 | 0.3896 | 0.5845 | 2.872 | 0.7288 |
|  |  |  |  |  |  |  |
| **Liver IL-1b** |  |  |  |  |  |  |
| Minimum | 0.4172 | 1.403 | 0.4144 | 1.557 | 2.556 | 0.7732 |
| Maximum | 2.233 | 50.53 | 5.148 | 50.88 | 31.76 | 4.921 |
| Range | 1.816 | 49.12 | 4.733 | 49.32 | 29.2 | 4.148 |
| Mean | 1 | 20.42 | 2.014 | 19.55 | 17.73 | 2.127 |
| Std. Deviation | 0.538 | 16.68 | 1.489 | 16.6 | 9.027 | 1.235 |
| Std. Error of Mean | 0.1701 | 5.275 | 0.471 | 5.249 | 2.854 | 0.3907 |
|  |  |  |  |  |  |  |
| **Liver TLR4** |  |  |  |  |  |  |
| Minimum | 0.7004 | 1.178 | 1.178 | 1.107 | 1.767 | 0.7718 |
| Maximum | 1.435 | 3.141 | 1.823 | 3.473 | 3.894 | 4.535 |
| Range | 0.7348 | 1.963 | 0.645 | 2.366 | 2.127 | 3.764 |
| Mean | 1 | 2.548 | 1.441 | 1.97 | 2.819 | 2.733 |
| Std. Deviation | 0.2103 | 0.6273 | 0.1947 | 0.9321 | 0.7653 | 1.166 |
| Std. Error of Mean | 0.07011 | 0.2091 | 0.06883 | 0.2948 | 0.242 | 0.3688 |
|  |  |  |  |  |  |  |
| **Liver TLR7** |  |  |  |  |  |  |
| Minimum | 0.5828 | 0.8532 | 1.284 | 1.334 | 1.44 | 1.215 |
| Maximum | 1.587 | 5.173 | 3.761 | 5.468 | 6.195 | 4.35 |
| Range | 1.004 | 4.32 | 2.476 | 4.134 | 4.755 | 3.135 |
| Mean | 1 | 3.046 | 1.912 | 3.072 | 2.791 | 2.71 |
| Std. Deviation | 0.3087 | 1.474 | 0.7901 | 1.727 | 1.453 | 1.095 |
| Std. Error of Mean | 0.09762 | 0.466 | 0.2498 | 0.5757 | 0.4593 | 0.3462 |
|  |  |  |  |  |  |  |
| **Liver SAA1** |  |  |  |  |  |  |
| Minimum | 1.648 | 153.9 | 79.95 | 0.4463 | 215.4 | 113.5 |
| Maximum | 83.63 | 669.1 | 216.9 | 2.782 | 844 | 346.3 |
| Range | 81.99 | 515.2 | 137 | 2.336 | 628.5 | 232.9 |
| Mean | 25.15 | 467.1 | 156.8 | 1 | 504.5 | 227.9 |
| Std. Deviation | 29.55 | 175.5 | 50.04 | 0.6603 | 187.6 | 80.02 |
| Std. Error of Mean | 9.345 | 55.51 | 15.82 | 0.2088 | 59.33 | 25.3 |
|  |  |  |  |  |  |  |
| **Liver SAA2** |  |  |  |  |  |  |
| Minimum | 0.4629 | 172.9 | 161.3 | 0.1182 | 242.8 | 121.4 |
| Maximum | 35.72 | 756.8 | 279.9 | 2.628 | 1645 | 509.8 |
| Range | 35.26 | 583.9 | 118.6 | 2.51 | 1402 | 388.4 |
| Mean | 6.658 | 507.9 | 211 | 1 | 839.1 | 328.7 |
| Std. Deviation | 12.12 | 162.9 | 38.47 | 0.7655 | 392.3 | 126.3 |
| Std. Error of Mean | 4.285 | 54.3 | 12.82 | 0.2421 | 130.8 | 42.09 |
|  |  |  |  |  |  |  |
| **Liver SAA3** |  |  |  |  |  |  |
| Minimum | 0.4737 | 360.1 | 431.2 | 0.2896 | 480.1 | 345.4 |
| Maximum | 2.095 | 3332 | 1065 | 3.415 | 6687 | 1022 |
| Range | 1.621 | 2972 | 634.2 | 3.126 | 6207 | 676.5 |
| Mean | 1 | 2081 | 730.8 | 1.412 | 3639 | 700.8 |
| Std. Deviation | 0.5364 | 998.4 | 241.9 | 1.059 | 1719 | 212.6 |
| Std. Error of Mean | 0.1696 | 315.7 | 76.49 | 0.335 | 543.7 | 67.23 |
|  |  |  |  |  |  |  |
| **PFC CXCL1** |  |  |  |  |  |  |
| Minimum | 0.685 | 0.6303 | 1.646 | 0.4504 | 0.5984 | 1.184 |
| Maximum | 1.658 | 33.92 | 4.64 | 1.547 | 30.47 | 3.338 |
| Range | 0.9727 | 33.29 | 2.994 | 1.096 | 29.87 | 2.154 |
| Mean | 1.019 | 14.22 | 2.725 | 1 | 13.49 | 1.969 |
| Std. Deviation | 0.3106 | 10.02 | 1.091 | 0.3534 | 9.08 | 0.6404 |
| Std. Error of Mean | 0.1035 | 3.169 | 0.3451 | 0.1118 | 3.027 | 0.2025 |
|  |  |  |  |  |  |  |
| **PFC CXCL10** |  |  |  |  |  |  |
| Minimum | 0.7987 | 0.5361 | 22.48 | 0.47 | 0.8183 | 10.06 |
| Maximum | 2.299 | 19.1 | 173.7 | 2.371 | 71.79 | 190.1 |
| Range | 1.5 | 18.57 | 151.2 | 1.901 | 70.97 | 180.1 |
| Mean | 1.477 | 7.813 | 85.51 | 1 | 23.09 | 92.4 |
| Std. Deviation | 0.5818 | 5.441 | 53.34 | 0.5815 | 25.07 | 53.14 |
| Std. Error of Mean | 0.184 | 1.721 | 16.87 | 0.1839 | 7.927 | 16.8 |
|  |  |  |  |  |  |  |
| **PFC IL1B** |  |  |  |  |  |  |
| Minimum | 0.6785 | 1.007 | 0.7957 | 0.6855 | 1.223 | 0.793 |
| Maximum | 1.676 | 5.898 | 2.949 | 1.279 | 5.919 | 2.585 |
| Range | 0.9979 | 4.891 | 2.153 | 0.5937 | 4.696 | 1.792 |
| Mean | 1.161 | 3.479 | 1.645 | 1 | 3.136 | 1.359 |
| Std. Deviation | 0.3508 | 1.592 | 0.7241 | 0.1956 | 1.284 | 0.5654 |
| Std. Error of Mean | 0.1109 | 0.5036 | 0.229 | 0.06186 | 0.406 | 0.1885 |
|  |  |  |  |  |  |  |
| **PFC BDNF** |  |  |  |  |  |  |
| Minimum | 0.5613 | 0.5574 | 0.6272 | 0.6037 | 0.2443 | 0.4389 |
| Maximum | 1.862 | 1.431 | 1.044 | 1.719 | 1.326 | 0.8391 |
| Range | 1.301 | 0.8734 | 0.4167 | 1.116 | 1.082 | 0.4002 |
| Mean | 1.311 | 0.9336 | 0.7749 | 1 | 0.7501 | 0.6502 |
| Std. Deviation | 0.4401 | 0.2516 | 0.1365 | 0.2987 | 0.3031 | 0.1374 |
| Std. Error of Mean | 0.1392 | 0.07957 | 0.04316 | 0.09444 | 0.09585 | 0.04579 |
|  |  |  |  |  |  |  |
| **PFC TLR4** |  |  |  |  |  |  |
| Minimum | 0.638 | 1.33 | 1.195 | 0.577 | 1.199 | 1.312 |
| Maximum | 2.754 | 3.51 | 4.175 | 1.805 | 2.707 | 2.023 |
| Range | 2.116 | 2.18 | 2.98 | 1.228 | 1.508 | 0.7113 |
| Mean | 1.208 | 2.228 | 2.221 | 1 | 2.045 | 1.665 |
| Std. Deviation | 0.6092 | 0.621 | 1.029 | 0.375 | 0.4438 | 0.2397 |
| Std. Error of Mean | 0.1926 | 0.1964 | 0.3253 | 0.1186 | 0.1403 | 0.07579 |
|  |  |  |  |  |  |  |
| **PFC TLR7** |  |  |  |  |  |  |
| Minimum | 0.6785 | 1.181 | 1.046 | 1.114 | 0.8209 | 0.9933 |
| Maximum | 1.376 | 2.603 | 3.865 | 6.192 | 2.354 | 13.79 |
| Range | 0.6974 | 1.422 | 2.818 | 5.078 | 1.533 | 12.79 |
| Mean | 1 | 1.65 | 2.312 | 3.441 | 1.559 | 4.587 |
| Std. Deviation | 0.2532 | 0.408 | 0.8392 | 2.075 | 0.4912 | 4.536 |
| Std. Error of Mean | 0.08008 | 0.136 | 0.2654 | 0.6561 | 0.1553 | 1.434 |
